# Cholesterol and Cholesterol-Lowering Medications in COVID-19—An Unresolved Matter

**DOI:** 10.3390/ijms251910489

**Published:** 2024-09-29

**Authors:** Thomas Grewal, Mai Khanh Linh Nguyen, Christa Buechler

**Affiliations:** 1 School of Pharmacy, Faculty of Medicine and Health, University of Sydney, Sydney, NSW 2006, Australia; thomas.grewal@sydney.edu.au (T.G.); mngu0111@uni.sydney.edu.au (M.K.L.N.); 2 Department of Internal Medicine I, Regensburg University Hospital, 93053 Regensburg, Germany

**Keywords:** statin, LDL receptor, aminotransferase, albumin

## Abstract

Infections with severe acute respiratory syndrome coronavirus 2 (SARS-CoV-2) cause coronavirus disease 2019 (COVID-19), a disease with very heterogeneous symptoms. Dyslipidaemia is prevalent in at least 20% of Europeans, and dyslipidaemia before SARS-CoV-2 infection increases the risk for severe COVID-19 and mortality by 139%. Many reports described reduced serum cholesterol levels in virus-infected patients, in particular in those with severe disease. The liver is the major organ for lipid homeostasis and hepatic dysfunction appears to occur in one in five patients infected with SARS-CoV-2. Thus, SARS-CoV-2 infection, COVID-19 disease severity and liver injury may be related to impaired cholesterol homeostasis. These observations prompted efforts to assess the therapeutic opportunities of cholesterol-lowering medications to reduce COVID-19 severity. The majority of studies implicate statins to have beneficial effects on disease severity and outcome in COVID-19. Proprotein convertase subtilisin/kexin type 9 (PCSK9) antibodies have also shown potential to protect against COVID-19. This review describes the relationship between systemic cholesterol levels, liver injury and COVID-19 disease severity. The potential effects of statins and PCSK9 in COVID-19 are summarised. Finally, the relationship between cholesterol and lung function, the first organ to be affected by SARS-CoV-2, is described.

## 1. Introduction

Coronavirus disease 2019 (COVID-19) is a highly contagious disease caused by infection with the severe acute respiratory syndrome coronavirus type 2 (SARS-CoV-2) virus. In most cases, the indications of SARS-CoV-2 infection range from asymptomatic to mild, but in about 30% of SARS-CoV-2 positive hospitalisations, critical and life-threatening illness such as sepsis can occur [1]. Hence, a better understanding of the underlying mechanisms responsible for the heterogeneity in COVID-19 disease outcome could improve personalised treatment options.

A substantial number of host cell receptors for SARS-CoV-2 have been identified in recent years (reviewed in [2]). The angiotensin-converting enzyme 2 (ACE2) is considered the main host cell receptor for SARS-CoV-2 and is expressed in the lung but also in the kidney, liver and heart. Other proteins that can function as SARS-CoV-2 (co-) receptor include CD147, neuropilin receptors, scavenger receptor class B member 1 and several others reviewed previously [3]. Furthermore, the low-density lipoprotein (LDL) receptor has been shown to facilitate SARS-CoV-2 internalisation in the ocular cell line ARPE-19, and one can speculate about this lipoprotein receptor also serving as a host cell receptor for this virus in other cells and tissues [4]. While SARS-CoV-2 variants generally have a high affinity for lung/bronchial cells in the human lower airway, the Omicron variants that emerged more recently mostly infect nasal epithelial cells [5] and instead depend on endosomal routes for cell entry (see below).

Once bound to the cell surface, the virus then gains cell entry either through fusion with the plasma membrane or via clathrin-dependent or -independent endocytosis [3,6,7,8]. For the latter, endocytosed viruses then need to exit the endolysosomal compartment to reach the cytosol in order to initiate virus replication. The molecular mechanisms involving these steps and related to ACE2-mediated SARS-CoV-2 entry have received the most attention. After the binding of the viral spike (S) protein to ACE2 at the cell surface, the subsequent cleavage of the S protein at the S2′ cleavage site by transmembrane protease, serine 2 (TMPRSS2) is required for viral entry. This enables fusion of the viral membrane with the plasma membrane of the host cell and subsequent uncoating of the viral RNA [6]. In the event of TMPRSS2 deficiency, the cell internalises the virus complexed with ACE2 via clathrin-mediated endocytosis, followed by the cleavage at the S2′ site through the action of cathepsins along the endocytic pathway. Subsequently, the cleavage of the S protein and the dissociation of S1 and S2 result in the initiation of membrane fusion [6]. Compared to other SARS-CoV-2 variants, in particular the Omicron variants have been shown to depend on endosomal cathepsin L for infection [5].

This review article summarises the multiple roles of cellular cholesterol for SARS-CoV-2 infection and COVID-19 disease progression. Intriguingly, data on the association of dyslipidaemia and statin use on COVID-19 disease severity and outcome are discordant (Table 1). Studies linking low serum cholesterol levels with SARS-CoV-2 infectivity and severe COVID-19 cases are listed. On the other hand, various other studies could not find an association between serum cholesterol levels and COVID-19 disease severity (Table 1), and a summary of these studies is a novelty of the present review. Although it is widely accepted that underlying liver disease contributes to COVID-19 severity, limited attention has yet been paid to liver dysfunction also modifying plasma lipid and lipoprotein profiles and how this may relate to SARS-CoV-2 infection and COVID-19 disease progression. Finally, the role of cholesterol in lung function is shortly summarised, an aspect relevant for viral infection and disease outcome that is commonly not discussed in articles aiming to connect serum cholesterol levels and SARS-CoV-2 infection.

## 2. The Multiple Roles of Cholesterol for SARS-CoV-2 Infection and Propagation

Cholesterol plays an indispensable role in the life cycle of SARS-CoV-2 and affects a substantial number of molecular events during SARS-CoV-2 infection, propagation and release, which has been reviewed in detail [3,25,26,27,28]. For instance, increased uptake of cholesterol derived from LDL and high-density lipoproteins (HDL) improved SARS-CoV-2 infection, most likely due to alterations in the membrane lipid composition that favoured virus infection, as well as compromising immune cell functions [29]. Vice versa, pharmacological lowering of total and LDL-cholesterol levels reduced SARS-CoV-2 infection [30].

**Figure 1:**
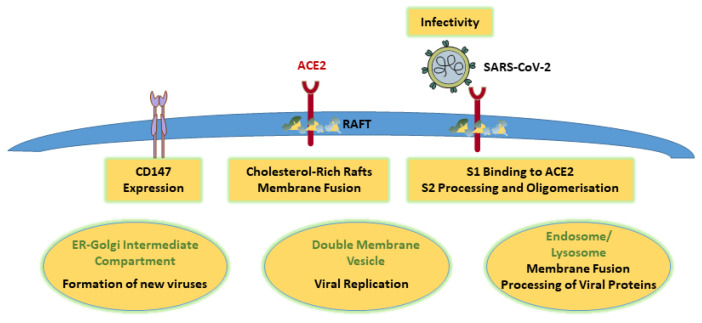
Cholesterol is essential for every step of the SARS-CoV-2 infection and replication cycle. All processes shown in yellow boxes or ellipses are disturbed upon cholesterol depletion. Removal of cholesterol from lipid rafts interferes with virus binding to ACE2 and inhibits fusion of the virus with the cell membrane, S1 binding to ACE2 and S2 processing and oligomerisation. Lowering cellular cholesterol levels may also reduce CD147 expression. Cholesterol removal also impairs fusion of the virus with the endosome/lysosome and processing of viral proteins in this compartment. Formation of double-membrane vesicles essential for viral replication is impaired. In the ER–Golgi intermediate compartment, low cholesterol prevents the building of virus particles. SARS-CoV-2 released from cells with low cholesterol is less infectious. Abbreviations: ACE2, angiotensin-converting enzyme 2; CD147: cluster of differentiation 147; ER, endoplasmic reticulum; S, spike.

Also, the association of ACE2 with cholesterol- and sphingolipid-rich specialised microdomains at the cell surface (lipid rafts) [31,32] is a prominent feature associated with efficient viral entry. This provided an opportunity to interfere with the docking and internalisation of the virus [2,33,34,35], and indeed, cholesterol depletion at the cell surface reduced S1 binding to ACE2 [2,36,37,38,39] (Figure 1). As ACE2 protein levels remained unchanged in the presence of cholesterol-depleting agents, ACE2 membrane localisation rather than ACE2 expression levels appeared crucial for viral entry [38]. Similarly, the novel compound 26,27-dinorcholest-5-en-24-yne-3β,20-diol (Nat-20(S)-yne) not only inhibited cholesterol synthesis but also blocked SARS-CoV-2 membrane fusion [40]. Furthermore, protein domains derived from bacterial cytolysins that specifically bind to membrane cholesterol provided antiviral effects, effectively inhibiting viral entry [41].

Besides ACE2, the transmembrane glycoprotein CD147 is one of the alternatives in the cellular repertoire that may serve as a receptor for SARS-CoV-2. Interestingly, cholesterol loading increased CD147 levels [42,43] (Figure 1), which may also serve to enhance the ability of SARS-CoV-2 to infect cells [6].

In addition to promoting endocytic pathways for viral entry, cholesterol upholds fusion of the viral envelope with lipid rafts. Several factors acting in a cholesterol-sensitive manner promoted fusion after S-protein docking onto ACE2, followed by TMPRSS2-mediated processing of the S2 subunit [44,45]. The latter was also stimulated by membrane cholesterol, which accelerated S2 activity through the formation of oligomeric S proteins [2,46,47,48] (Figure 1). Another link to cholesterol homeostasis was revealed by the activation of the master regulator of cellular cholesterol feedback control, sterol regulatory element-binding protein 2 (SREBP2), which enhanced the fusion of SARS-CoV-2 with the plasma membrane by optimising S2 processing [49].

Once entering the cell via the endocytic pathway [50,51], the virus ultimately reaches the late endosomal/lysosomal compartment, where an acidic pH of 6.2–6.8 for fusion and the release of its content is required. In this location, enveloped viruses including SARS-CoV-2 commonly capture late endosomal proteins to enter the cytoplasm and release the viral genome. For example, the late endosomal cholesterol transporter Niemann–Pick Type C1 (NPC1) aids the cell entry of Ebola and other filoviruses [52,53]. Vice versa, pharmacological inhibition of NPC1 strongly reduces infection with SARS-CoV-2 [54] and interferes with the fusion of viral envelopes with late endosomal membranes [55,56,57] (Figure 1). Other outcomes of blocking cholesterol export from endolysosomes include the impaired activity of cathepsins, interfering with cathepsin-dependent proteolytic processing for endocytosed virus to enter the host cell [35,58,59].

Further linking endolysosomal acidification with viral entry and NPC1, an increase in the acidic pH in this location also impaired S1 function, thereby protecting cells from infection. The binding of S1 to SLC38A9, an arginine sensor localised in endolysosomes, was critical for this regulatory circuit. SLC38A9 depletion impaired S1-associated deacidification in several cell lines, resulting in the inhibition of S protein-mediated entry of pseudo-SARS-CoV-2 [60]. SLC38A9 forms a complex with NPC1, which in turn activates the mechanistic target of rapamycin complex 1 (mTORC1) in a cholesterol-sensitive manner [61,62]. Thus, mTORC1 inhibition in the absence of SLC38A9 may be a strategy to impede the initial stages of SARS-CoV-2 infection [63].

Interestingly, depletion of another member of the NPC protein family, Niemann-–Pick C1 like 1 (NPC1L1), which is localised at the apical membrane of enterocytes to facilitate intestinal cholesterol absorption, reduced SARS-CoV-2 entry in human embryonic kidney cells [64]. NPC1L1 is inhibited by the cholesterol-lowering drug ezetimibe [65], which impaired viral entry and was associated with improved recovery in COVID-19 patients [66].

In addition, members of the oxysterol-binding protein (OSBP) family, which can transfer sterols across membranes in exchange for phosphoinositides, can influence viral replication. In particular, OSBP provides cholesterol from the endoplasmic reticulum to double-membrane vesicles (DMVs), essential replication organelles for many viruses, including SARS-CoV-2, thereby enhancing viral replication [67,68] (Figure 1). Moreover, pharmacological inhibition of OSBP revealed potent antiviral activity. Along these lines, blocking cytosolic phospholipase A2α, which produces lysophospholipids and requires cholesterol to promote vesiculation events, reduced DMV numbers and viral replication [69,70].

Furthermore, the formation of new viruses requires the establishment of spike trimers, which partition preferentially in cholesterol-rich domains in the ER–Golgi intermediate compartment (ERGIC), where the assembly of mature viruses occurs [71]. This is in line with earlier studies identifying budding from cholesterol-rich sites at the plasma membrane to provide lipid-containing viruses with a cholesterol-rich envelope [72]. In contrast, cholesterol depletion inhibited virus production, interfered with the structural integrity of the viral envelope [73] and, in some studies, could be rescued by cholesterol supplementation [25]. Finally, cholesterol levels in the SARS-CoV-2 virus envelope are critical for infectivity, as treatment of virus particles with methyl-beta-cyclodextrin impaired infection [47] (Figure 1). Likewise, cholesterol sequestration in endolysosomes and the concomitant decrease in cholesterol at the cell surface correlated with a reduced cholesterol content of influenza virus envelopes [74]. We speculate similar mechanisms can occur for the release of SARS-CoV-2 virus with consequences for viral infectivity.

Despite the many roles described above for cellular cholesterol modulating various steps during viral entry, propagation and exocytosis, pharmacological interventions targeting cholesterol-sensitive steps in the life cycle of SARS-CoV-2 will need a better understanding not only of cellular cholesterol homeostasis and functions but also how this may be influenced by overall physiology and disease-related alterations in the cholesterol levels of serum lipoproteins [75]. The relationships between serum cholesterol and lipoprotein levels with SARS-CoV-2 infection are summarised in the following sections.

## 3. Cholesterol-Containing Lipoproteins and Other Risk Factors Contributing to COVID-19 Severity

From epidemiological studies, it is long known that plasma cholesterol levels are affected by a plethora of diseases. In particular in the context of viral infections, there is a correlation between disease severity and serum cholesterol levels [76,77,78,79,80]. However, the underlying changes in lipid metabolism driven by SARS-CoV-2 infection are not fully understood and require further insight into how the viral infection develops over time, how infection of different tissues impacts on whole body cholesterol homeostasis and if other risk factors influence these parameters.

SARS-CoV-2 initially infects pulmonary cells, which can cause a hyperinflammatory stage leading to damage of other tissues and organs. Moreover, SARS-CoV-2 may also infect non-pulmonary cells such as cholangiocytes and hepatocytes, and this was proposed to contribute to the wide range of liver-related COVID-19 symptoms [81]. However, the route that allows SARS-CoV-2 to enter the liver to infect hepatocytes remains to be clarified [82].

**Figure 2:**
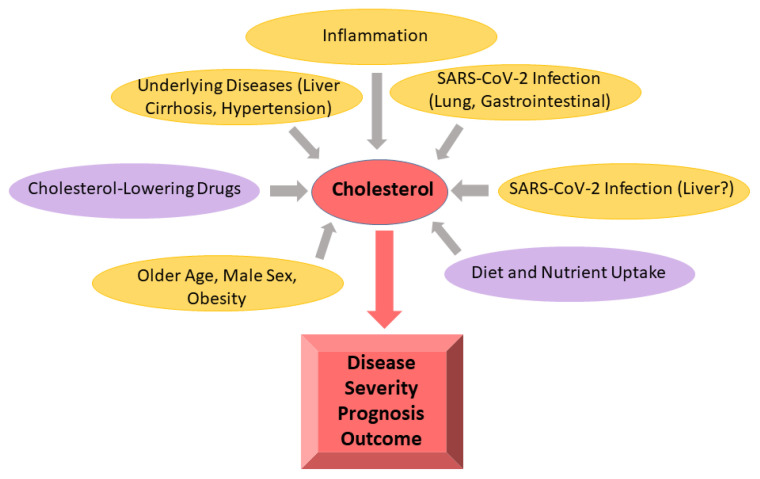
Serum cholesterol levels are related to systemic inflammation, underlying diseases, intake of cholesterol-lowering drugs as well as gender, age and obesity. SARS-CoV-2 infection may affect serum cholesterol levels, and the type of the infected organs may have a role herein. Severe illness may limit nutrient intake and lead to low serum cholesterol levels. Conditions that contribute to increased SARS-CoV-2 severity are shown in yellow and those that are protective are shown in pink. It should be noted that a protective effect of cholesterol-lowering statin therapy and an association of blood cholesterol with COVID-19 disease severity is still an unresolved issue (see text for details).

The liver is the main organ in the control of lipoprotein and cholesterol metabolism. This ranges from the release of very-low-density lipoprotein (VLDL) to LDL clearance and HDL-mediated reverse cholesterol transport for bile excretion [83,84]. COVID-19 severity was found to be associated with the gravity of liver disease [85]. Moreover, patients with underlying liver cirrhosis, who have low serum cholesterol levels in addition to other severe dysfunctions [77,86], have a higher risk for COVID-19 mortality [87] (Figure 2). While these observations may indicate the marker potential of low serum cholesterol levels for COVID-19 severity in some patients with liver dysfunction, aspartate and alanine aminotransferases (AST, ALT), alkaline phosphatase (AP) and bilirubin are used for assessing the condition of the liver. ALT and AST are markers of acute liver injury caused by viral infection or toxins. While the determination of ALT levels in plasma indeed mostly reflects liver function, it is now well recognised that systemic AST levels can also increase when other organs are damaged. In addition, levels of these enzymes can be induced in respiratory and/or gastrointestinal viral infections, which are referred to as nonspecific reactive hepatitis [88]. Gamma-glutamyltransferase levels are used as a marker for hepatobiliary diseases and are a prognostic biomarker in severely ill patients [89]. Elevated levels of all of these enzymes may indicate acute liver injury, drug-induced hepatic injury, muscle breakdown and inflammation, and assessment of liver synthetic function markers such as albumin or international normalised ratio may be more appropriate for the diagnosis of liver disease related to SARS-CoV-2 infection [90].

Hence, studies evaluating the association of serum cholesterol levels with disease severity in SARS-CoV-2 infection need to consider whether the above-listed markers for impaired liver function correlate with or even contribute to lower plasma sterol levels. However, commonly used markers of liver health such as ALT and AST are also induced by inflammation and have limited suitability as sole markers when assessing liver function in COVID-19 patients [90]. Thus, associations between low plasma cholesterol levels and COVID-19 severity may be confounded by liver disease and other factors, complicating predictions for COVID-19 disease outcome and survival [91,92,93].

Like the links of ALT and AST enzymes with inflammation, lipoprotein levels are unlikely to solely reflect liver function in COVID-19 patients, as lipids and lipoproteins are well described for their immune-regulatory functions. HDL mostly exerts anti-inflammatory and antioxidant effects. The anti-inflammatory activity of HDL does not correlate with HDL-cholesterol levels and should rather be estimated by its ability to suppress tumour necrosis factor activity in endothelial cells [94]. On the other hand, low HDL-cholesterol levels increase the risk of developing an infection and sepsis [95,96].

LDL was mostly shown to exert pro-inflammatory activities [97]. In particular, oxidative modification of LDL generates an inflammatory and proatherogenic particle. Oxidised LDL is produced in excess in inflammatory and infectious diseases, increasing the risk for cardiovascular disease [98]. In contrast, LDL can bind LPS, thereby reducing its biological activity, and mice lacking the LDL receptor were indeed protected from endotoxaemia [99].

Altered lipoprotein levels also relate to advanced age and male sex, two major predictors of adverse outcomes in SARS-CoV-2-infected patients [100,101] (Figure 2). In some Western world countries, 25–30% of young males and females already display elevated plasma cholesterol levels, and hypercholesterolaemia prevalence increases with age in both sexes [102]. Accordingly, a high percentage of the European and North American population are prescribed statins [103]. Elevated systemic levels of total cholesterol are more prevalent in women, and HDL levels in males are lower compared to women of all ages [102]. This indicates that sex-specific analysis of cholesterol levels is needed to better evaluate the relationship between systemic cholesterol levels, lipoprotein profiles and COVID-19 severity.

Obesity is another risk factor for severe COVID-19 disease and adverse outcomes [104], with dyslipidaemic lipoprotein profiles being common in approximately 60–70% of the obese. With regard to plasma cholesterol levels, obesity coincides with low HDL-cholesterol levels, while LDL-cholesterol levels are normal or modestly increased [76] (Figure 2). The overweight/obese more often suffer from comorbidities such as type 2 diabetes, hypertension, nonalcoholic fatty liver disease and cardiovascular diseases, all associated with an increased risk for severe COVID-19 [105].

The latter predictors for alterations in lipid profiles in the human population, age, sex and obesity highlight the difficulty to establish levels of plasma cholesterol or other lipids as a predictor for COVID-19 disease outcome. In fact, dyslipidaemia is prevalent in the general population, from which up to 60% may be affected [102]. A retrospective study reported that dyslipidaemia before hospital admission was related with worse COVID-19 disease outcome [13]. In one of the cohorts analysed, high LDL-cholesterol levels were related to recurrence of SARS-CoV-2 positivity after discharge. In the second cohort, high triglycerides were associated with mortality [13]. Similarly, a meta-analysis of seven studies with approximately 6900 patients revealed an increased risk for severe COVID-19 in patients with dyslipidaemia [12]. An association of dyslipidaemia with severe COVID-19 was also identified by another meta-analysis including nine studies and more than 3650 patients. This relationship was stronger in males, older patients and patients with hypertension [106]. Increased HDL levels before SARS-CoV-2 infection were related to a lower risk for SARS-CoV-2 infection and reduced risk of death [9,10]. After adjusting for age, sex, obesity, hypertension, type 2 diabetes and coronary artery disease, an increase in serum HDL-cholesterol levels of 10 mg/dl was linked to a 10% decreased risk of SARS-CoV-2 infection [9]. Likewise, higher HDL-cholesterol levels prior to COVID-19 disease were related to a lower risk of death [107]. On the other hand, others reported similar baseline total, HDL- and LDL-cholesterol and triglycerides levels of patients with nonsevere and severe disease and nonsurvivors and survivors [14]. In line with this, hypercholesterinaemia in healthcare workers was not associated with a higher SARS-CoV-2 infection rate [11].

During COVID-19 disease, associations of systemic cholesterol, LDL and HDL levels with COVID-19 severity and outcome have been described in some but not all studies. As outlined above, the beneficial effects of cholesterol-lowering drugs have also been reported in many research studies. In the following two sections, we will summarise studies addressing the relationship between cholesterol levels and COVID-19 disease severity.

## 4. The Association of Low Plasma Cholesterol Levels with Severe COVID-19

Critically ill patients are often reported to display low circulating cholesterol levels, an observation also reported for patients with severe COVID-19 [3,17,108].

Given that serum cholesterol levels commonly reflect continuing dietary changes, the inappropriate or compromised uptake and metabolism of nutrients by severely ill patients may be one factor contributing to low serum cholesterol levels (Figure 2). In contrast, in critical illness, triglyceride levels remained unchanged or were even increased due to enhanced lipolysis in fat tissues and impaired mitochondrial beta-oxidation [109]. This was also reflected in patients with severe COVID-19 where both LDL- and HDL-cholesterol amounts declined, while triglyceride levels continued to be normal or only showed a tendency to rise [17,110].

Lowered concentrations of cholesterol-rich lipoproteins in COVID-19 correlated with the severity of the underlying disease, and HDL-cholesterol, its major apolipoprotein A-1 (ApoA-1) and total cholesterol levels were predictive of mortality [15]. An association of COVID-19 disease severity with altered lipoprotein metabolism and reduced plasma cholesterol levels was supported by the higher systemic lipids of patients hospitalised in a normal ward compared to patients admitted to intensive care [111]. A meta-analysis assessing data from 29 studies with ~260,000 participants further highlighted COVID-19 patients to have lower HDL- and total cholesterol levels compared to noninfected controls. Furthermore, severely affected COVID-19 and deceased patients exhibited lower LDL-, HDL- and total cholesterol concentrations. This meta-analysis did not reveal any changes in serum triglyceride levels upon SARS-CoV-2 infection, and circulating triglyceride concentrations were also not associated with disease severity or mortality [17].

Pointing at the prognostic value of lipoprotein cholesterol levels for disease outcome, low LDL- and HDL-cholesterol amounts in COVID-19 patients were associated with a more severe disease course and death [16]. Stratification of 211 severe and critical COVID-19 patients according to their LDL-cholesterol levels showed that patients with the lowest amounts of LDL cholesterol had a higher risk for admission to the intensive care unit and death compared to patients with higher plasma concentrations of LDL cholesterol [112].

Interestingly, according to several studies, comorbidities and liver injury only contributed marginally to systemic cholesterol levels in COVID-19 patients. Patients stratified for LDL concentrations had a similar prevalence of comorbidities such as diabetes, acute liver and renal injury. COVID-19 patients with the lowest LDL-cholesterol levels had modestly higher AST amounts, whereas ALT and bilirubin levels were in the normal range and estimated glomerular filtration rate was low and comparable in all LDL-cholesterol categories. C-reactive protein (CRP) and procalcitonin levels, which are commonly used as inflammation markers, did not increase with lower LDL-cholesterol concentrations in COVID-19 cohorts [112,113]. Thus, this analysis could not prove an association between low systemic cholesterol levels and inflammation. The study also suggested that underlying diseases affecting circulating cholesterol levels such as liver diseases and renal injury [77,114] did not greatly contribute to low serum cholesterol concentrations observed in COVID-19 patients. Accordingly, while serum levels of AST, ALT, AP and GGT remained unchanged, the amounts of LDL, HDL and total cholesterol, but also triglycerides, declined with increasing COVID-19 disease severity [111].

As mentioned earlier, men are at higher risk for a more severe disease course upon infection with SARS-CoV-2 [115,116], an association that persists even after controlling for confounding factors such as comorbidities [116]. Given that (i) serum lipids differed between sexes, (ii) menopause led to lipid profile changes [117] and (iii) there was a gender-specific association of serum lipid levels with disease biomarkers [118,119,120], it was speculated that these differences in lipid metabolism, including lipoprotein-associated cholesterol levels, may contribute to more serious COVID-19-related illness in men.

However, there are currently limited data to support this hypothesis, as the majority of COVID-19-related information on serum lipid levels did not account for gender-specific factors. In a recent study, like plasma cholesterol levels, metabolic profiling of males and postmenopausal females identified specific lysophospholipids, plasmalogens and lysoplasmalogens to decline with COVID-19 severity, yet the decrease in these particular lipids was more pronounced only in male COVID-19 patients. In contrast, these lipid species did not reveal gender-related differences in the control cohort [121]. The physiological significance of male-specific diminishments of these lipid classes in severe COVID-19 disease remains unclear. Lysophospholipids are highly abundant in plasma and constituents of lipoproteins while serving as membrane building blocks and signalling molecules in cells [122,123]. Furthermore, through the binding to various lysophospholipid receptors, these lipids modulate the functioning of immune cells [124,125]. Plasmalogens are considered to protect from oxidative stress and inflammation, and their reduced levels in male patients with severe COVID-19 may contribute to an enhanced inflammatory response [121,126]. Of note, COVID-19-positive males had higher levels of primary and secondary bile acids in comparison to COVID-19-infected females [121]. While elevated amounts of bile acids were also observed in noninfected males vs. females, the role of bile acids in nutrient absorption, the functioning of the intestinal microbiome and their ability to act as inflammatory lipids may also contribute to disease outcome [127].

Serum levels of primary and secondary bile acids increased with COVID-19 disease severity and normalised in mild COVID-19 when patients recovered [128]. Bile acids were described to protect against COVID-19 by regulating the expression of ACE2 and the inflammatory response [129]. To our knowledge, the levels of fecal bile acid and fecal cholesterol levels in COVID-19 patients is still unknown, and thus it remains unclear whether lower serum cholesterol levels described in some cohorts with severe COVID-19 disease [17,28] can be attributed to enhanced biliary excretion of bile acids.

Although the underlying mechanisms causing hypocholesterolaemia in critical illness are not completely understood [108], a higher demand for cholesterol in infected cells to support microbial propagation has been considered as a critically contributing factor. In line with this hypothesis, humans and rodents exposed to lipopolysaccharide (LPS) displayed a decline in circulating lipids, pointing at inflammation having a significant role in the development of hypocholesterolaemia upon bacterial and viral infections [130]. Both moderate and severe COVID-19 patients had significantly lower serum concentrations of cholesterol and the precursor steroids lanosterol, desmosterol and lathosterol than controls, indicating impaired cholesterol biosynthesis. The decline in these sterols was not related with disease severity [131].

Yet, related disease phenotypes caused by dissimilar infections may still differ in their impact on plasma cholesterol levels. Comparison of the blood lipidome of patients with community-acquired pneumonia (CAP) and COVID-19 pneumonia patients revealed lower total and LDL-cholesterol levels in the latter group. Both patient cohorts had elevated levels of atherogenic lipoproteins such as VLDL and intermediate-density lipoprotein. The decline in cholesteryl ester levels was observed in both cohorts, and free cholesterol was lower in patients with COVID-19 [132].

Strikingly, during recovery from SARS-CoV-2 infection, LDL-, HDL- and total cholesterol levels increased 3–6 months after hospital discharge, showing normalisation of lipid levels [133]. On the other hand, patients with post- or long-COVID-19 symptoms appear at a higher risk for dyslipidaemia compared to noninfected controls, showing increased LDL-cholesterol and triglyceride levels and lower HDL-cholesterol concentrations [15,134].

## 5. Studies Challenging the Association of Low Plasma Cholesterol Levels and COVID-19 Severity

Notably, while the above-listed studies generally favour an association of low plasma cholesterol levels with COVID-19 severity, several other studies did not support this hypothesis. For instance, analysis of asymptomatic COVID-19 patients revealed normal cholesterol levels, excluding that virus infection alone was the driver to impact plasma lipid levels [135]. In another study, COVID-19 patients (n = 100) displayed normal HDL, LDL and triglyceride levels in comparison to healthy controls, only showing an increase in oxidised LDL levels. Markers for liver dysfunction including albumin, AST, ALT and bilirubin were also in the normal range [136]. Likewise, systemic triglycerides and cholesterol levels from COVID-19 patients (n = 97) were not related to in-hospital mortality [19]. A comparison of mild (n = 1362) and severe (n = 127) COVID-19 patients described similar total cholesterol levels. In this cohort, severe disease was accompanied by low HDL-cholesterol, low albumin and high AST and ALT levels. This analysis suggested that reduced liver synthesis function was not associated with lower LDL-cholesterol levels [20]. Another study reported cholesterol and triglyceride levels to be similar in nonhospitalised, normal ward and intensive care COVID-19 patients. ALT and AST but not GGT levels increased with COVID-19 disease severity, and hospitalised patients had high CRP levels [137]. Similarly, cholesterol, triglyceride, HDL and LDL levels were comparable in COVID-19 patients and healthy controls. COVID-19 patients from this cohort presented increased CRP as well as ALT and GGT levels, a reduced international normalised ratio and lower albumin concentrations, indicating impaired hepatic synthesis function, whereas bilirubin was normal [18]. A two-sample Mendelian randomisation approach indicated that the relationship between COVID-19 and dyslipidaemia reflected the exacerbation of underlying comorbidities rather than COVID-19 severity [138]. In line with that study, the comparison of COVID-19 patients with and without hypertension revealed that several of the previously described changes in lipid metabolites of patients with COVID-19 could be related to high blood pressure [139] (Figure 2).

Both LDL and HDL particles contain cholesteryl esters in their particle cores, and lipidomic profiling of cholesteryl ester species (n = 22) in serum from severe and mild COVID-19 cases did not reveal any significant differences [140] but rather implicated dysregulation of di- and triacylglycerol metabolism and elevated ceramide species in the serum of patients with more severe COVID-19 disease [140]. Yet, the diversity of cholesteryl ester species might obscure differential effects on certain lipid classes and might be related to COVID-19, as a previous study from our group of 15 cholesteryl ester species in the serum of patients with moderate and severe COVID-19 showed that most of the cholesteryl ester classes with shorter fatty acid chains decreased in severe compared to moderate COVID-19. Within the two cohorts, free cholesterol levels increased with COVID-19 severity, whereas total serum cholesterol levels of patients with moderate and severe disease were comparable. Cholesteryl ester species negatively correlated with CRP in severe COVID-19 patients [141]. The free cholesterol/cholesteryl ester ratio correlated positively with CRP, procalcitonin, IL-6 and ferritin in moderate and severe COVID-19, suggesting that impaired esterification of cholesterol rather than total serum cholesterol levels was related to systemic inflammation and disease severity in patients with COVID-19. Of note, patients with liver cirrhosis that are commonly characterised by low serum cholesterol levels were excluded from the analysis [141]. Yet, given the limited knowledge on the function of these different cholesteryl ester species, the pathophysiological consequences of altered lipid class composition in COVID-19 needs further study.

In summary, the association of LDL-, HDL- and total serum cholesterol levels with COVID-19 disease severity and outcome has not been finally resolved. Most of the studies listed above consist of rather small cohorts and cannot account for confounding factors such as underlying diseases, comorbidities, drug use, COVID-19 related liver injury, renal dysfunction and sex. Nevertheless, given the plentiful implications of cellular cholesterol depletion interfering with viral infection and propagation, statins and other cholesterol-modifying drugs have been analysed for their potential use to combat SARS-CoV-2 infection and COVID-19, which is summarised in the following section.

## 6. Statins Offer Multiple Modes of Action with the Potential to Lower COVID-19 Severity

Statins are commonly used drugs that block 3-hydroxy-3-methylglutaryl-coenzyme A (HMG-CoA) reductase, catalysing an early step in cholesterol biosynthesis. Subsequent activation of the feedback control that governs cholesterol homeostasis then leads to the upregulation of LDL-receptor expression, which promotes hepatic clearance of LDL, effectively lowering systemic LDL-cholesterol levels [142].

**Figure 3:**
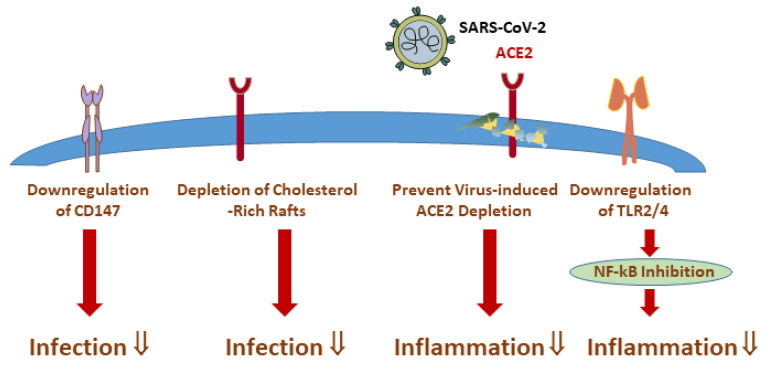
Pleiotropic effects of statins in COVID-19 disease. Statins remove cholesterol from lipid rafts, which interferes with virus binding to ACE2. Statins downregulate CD147, which is also involved in virus internalisation. Statins interfere with the processing of viral proteins. Statins prevent infection-induced downregulation of ACE2, which is thought to protect against inflammation. Statins reduce TLR2/4 expression and inhibit NF-kB activity. Whether the cholesterol-lowering effect of statins is still significant in critical illness needs further study. Abbreviations: ACE2, angiotensin-converting enzyme 2; CD147, cluster of differentiation 147; NF-kB, nuclear factor kappa-light-chain-enhancer of activated B cells; TLR2/4, toll-like receptor 2/; ⇓, reduction.

At the cellular level, statins lower the cholesterol content of the plasma membrane, thereby reducing the number of cholesterol-rich microdomains, which are essential for viral entry [143,144,145] (Figure 3). This activity has been described for the lipophilic statins such as lovastatin, simvastatin, pitavastatin and atorvastatin [2]. An underlying cause for this observation appears to be the reduced association of ACE2 with lipid rafts, which is required for efficient viral docking [38,144,145]. After ACE2-mediated SARS-CoV-2 entry, successive ACE2 downregulation contributes to an overactivation of the renin-angiotensin system, which controls blood pressure, electrolyte balance and systemic vascular resistance. Yet, statin-mediated activation of the transcriptional network of the cholesterol feedback control is probably responsible for the elevation of ACE2 levels [145,146] (Figure 3), thereby reducing the risk of vasoconstriction, inflammation, oedema and fibrosis that contribute to COVID-19 severity [147,148].

In addition, prolonged treatment with both hydrophilic and lipophilic statins downregulated TMPRSS2 expression in a dose-dependent manner, ultimately reducing SARS-CoV-2 processing after docking to ACE2, compromising the fusion of the virus with the plasma membrane [145].

Other beneficial effects of pravastatin, fluvastatin and atorvastatin include their ability to suppress cell surface expression of CD147, which can function as an alternative SARS-CoV-2 receptor [144,149] (Figure 3). This observation can be explained by statins inhibiting the conversion of 3-hydroxy-3-methylglutaryl coenzyme A to mevalonic acid, which serves as a precursor for a multitude of pathways unrelated to cholesterol synthesis, such as isoprenylation and *N*-glycosylation of proteins. The loss of these post-translational modifications upon statin exposure was responsible for altered CD147 expression, structure and function in THP1 macrophages [149].

Other properties of statins that may contribute to their ability to reduce COVID-19 severity include their anti-inflammatory activities. These findings can in part be elucidated by statins compromising the mevalonate-dependent production of isoprenoid intermediates such as farnesylpyrophosphate and geranylgeranyl-pyrophosphate. These metabolites are required for the membrane anchoring of small signalling proteins of the Ras superfamily that play prominent roles in inflammation and are anchored at the plasma membrane. Statin-mediated lowering of isoprenoid synthesis and loss of prenylation of these signalling proteins ultimately reduced activation of various pathways that drive inflammatory response [150,151].

In addition, statins lowered the expression of the toll-like receptors 2 and 4 and thereby protected immune cells from a wide range of ligands originating from viruses, fungi and Gram-negative and Gram-positive bacteria. Statins also inhibited the activity of the transcription factor nuclear factor-kappa B (NF-kB) activity (Figure 3) [150,152] and lowered inflammation of SARS-CoV-2-infected lung tissues, human monocytes and neutrophils [145,146]. The underlying mechanisms that enable statins to downregulate NF-kB are not fully understood, but many cell- and animal-based models and data from humans implicated atorvastatin, fluvastatin, lovastatin, pravastatin and simvastatin to facilitate anti-inflammatory effects via NF-kB inhibition [150,153]. One hypothesis proposed the ability of statins to scavenge free oxygen radicals and stimulate nitric oxide production, which both stabilise the NF-kB inhibitor protein [154,155].

A significant number of severely ill COVID-19 patients develop thrombotic complications despite anticoagulation. Remarkably, statins have pleiotropic antithrombotic effects, and besides the reduction of Rho-GTPase and NF-kB activity and reduced pro-inflammatory cytokine production, this includes lowering of oxidative stress, decreasing the activity of the coagulation cascade, as well as downregulating prothrombin activation and thromboxane A2 levels (reviewed in [156]).

Finally, a number of in silico molecular docking studies proposed several statins to bind multiple SARS-CoV-2 proteins and SARS-CoV-2 receptors (Figure 3). Statins were proposed to bind the main protease (M^pro^) of SARS-CoV-2 involved in the processing of viral proteins [157], the S protein, viral helicase and RNA-polymerase [158] (Figure 3). While these computational predictions have the potential to identify novel mechanisms mediated by statins, the lack of lab-based validation studies still limits the antiviral and therapeutic potential of these observations.

## 7. Statins Influence COVID-19 Severity and Outcome

Most patient-related studies addressing the relationship between statin therapy and COVID-19 disease outcome did not provide information about serum cholesterol, lipoprotein and apolipoprotein levels. Thus, a clear relationship between statin intake and serum cholesterol in COVID-19 still has to be established. Yet, given the wide range of COVID-related disease progression and symptoms, together with the variation in statin efficacy in patients, this remains a challenge. In addition, the cholesterol-lowering effects of statins are disrupted in an inflammatory environment, a state called statin resistance [159,160], which has to be considered when interpreting current and future studies.

In addition to the challenges listed above, another challenge further complicating the analysis of patient data relates to the differential pharmacokinetics and -dynamics of statins that might vary over the course of COVID-19. The mean concentration of statins in human serum ranges from 1 to 15 nM, and considering that most of these drugs are protein-bound in the blood, the free, active fraction of statins can differ greatly, varying from 0.01 to 0.5 nM. Similarly, the dosage in patients probably varies in the range of 0.1–1 mg/kg body weight, whereas 1–500 mg/kg body weight has been employed in the majority of rodent studies [161]. Furthermore, at least in cell-based studies, contrasting activities can be achieved by high and low doses of statins [161]. This extends to inflammatory settings, with low and high doses of statins exerting differential effects on NF-kB activity in the presence or absence of LPS [145]. Therefore, care needs to taken when extrapolating results from cell-based or rodent studies to patients without taking into account the above limitations.

In the following, we list studies that aimed to correlate statin use with COVID-19 severity. In fact, to date, an enormous number of epidemiological and lab-based studies aimed to clarify if statins can be associated with improved COVID-19 disease outcomes. From these studies, it still remains difficult to correlate statin treatment not only with COVID-19 severity but also with systemic cholesterol levels, as most reports suggest that the protective effect of statins with regard to SARS-CoV-2 infections is related to their anti-inflammatory effects [162].

There is evidence that statin therapy can increase ApoA-I levels in patients. Pitavastatin therapy of hypercholesterolaemic patients increased ApoA-I levels. This effect was much less pronounced with atorvastatin despite a comparable effect of both drugs on LDL, HDL and CRP levels [163]. In a murine endotoxaemia model, ApoA-I protected from inflammation and lung injury [164], and increased ApoA-I levels of statin-treated COVID-19 patients may provide a further beneficial effect of statins.

Unlike the anti-inflammatory action proposed for statins in COVID-19, drugs that mostly lowered inflammation were not effective in patients with severe illness [165,166]. Inflammation is a crucial element in the activation of both the innate and the adaptive immune systems due to the role of inflammatory cytokines in this process. The blockade of this pathway is detrimental to the treatment of sepsis, and anti-inflammatory therapies correlate with a higher risk of infection. Glucocorticoids are commonly used for the treatment of inflammation despite the lack of evidence supporting a survival advantage [166]. Hence, the beneficial effects of statins in severe COVID-19 are unlikely to be limited to their anti-inflammatory properties but rather may be multifactorial and involve other mechanisms and pathways.

Notably, the outcomes of many cohort studies were often in doubt of promoting statins as an antiviral strategy against COVID-19, in particular findings from meta-analyses of retrospective studies. The interpretation of statin-related patient data remains difficult, as statins are commonly taken by patients with cardiovascular risks and complications. These patients have a higher risk for severe COVID-19, and observational studies have to be corrected for multiple confounding variables [150]. Nevertheless, studies accounting for various covariates favoured a beneficial effect of statin use [23]. A recent meta-analysis showed that statin use significantly reduced mortality, admission to intensive care and the need for mechanical ventilation when pooling adjusted odds ratios [23]. Patients who were given statins after their COVID-19 diagnosis had a lower mortality risk, and among patients not admitted to intensive care, patients on statins had a lower mortality rate compared to nonstatin users [22]. Preadmission statin treatment was also found associated with better outcomes among COVID-19 patients with a high to very high cardiovascular risk [21]. Statin treatment protected from death in a cohort of >3000 patients and the subgroup of patients with coronary heart disease [167]. Furthermore, in COVID-19 patients with type 2 diabetes, statins reduced the risk of developing chronic cough and dyspnoea [168].

On the other hand, it was also reported that statin use before COVID-19 hospitalisation did not protect from fatal outcomes [24], and patients taking statins exhibited the same risk of infection with SARS-CoV-2 as nonusers [169]. Statin-user prevalence was higher in severe than in mild COVID-19 disease and in nonsurvivors than in survivors [20]. As mentioned above, statin doses may also make a difference, as only low and moderate but not high doses of statin reduced the risk of hospitalisation of COVID-19 patients compared with nonusers [170]. Rhabdomyolysis is often reported in hospitalised COVID-19 patients who take statins and highlights that statin-related side effects also need to be considered [171].

In patients with acute respiratory distress syndrome triggered by sepsis, a modestly improved survival rate occurred in patients using lipophilic simvastatin, while hydrophilic rosuvastatin was associated with increased mortality [172]. Hence, the differential effects of hydrophilicity and lipophilicity of statins [173] seem to matter for certain COVID-19 disease outcomes.

Altogether, based on the majority of the abovementioned studies, statin use appears to have a protective effect on COVID-19 severity and outcome. Further research is required to better identify the COVID-19 patients who will benefit from statin use.

## 8. PCSK9 and COVID-19 Disease

LDL-derived cholesterol is taken up by cells through LDL receptor-mediated endocytosis. Once the LDL/LDLR complex reaches the endosomal sorting compartment, LDL particles dissociate from the LDL receptor, the latter being recycled back to the cell surface for another round of LDL endocytosis. In spite of this, the systemic proprotein convertase subtilisin/kexin type 9 (PCSK9) can bind to the LDL receptor, which is then targeted to lysosomal degradation, thereby reducing the number of LDL receptors at the cell surface. Thus, elevated PCSK9 levels or PCSK9 gain-of-function mutant carriers exhibit reduced LDL particle clearance from plasma, ultimately increasing circulating LDL-cholesterol levels [142,174]. Inhibitory PCSK9 monoclonal antibodies have been developed and are used for the treatment of hypercholesterinaemic, statin-intolerant patients [142]. Interestingly, while statin-mediated LDL-receptor upregulation lowers circulating LDL levels, statins also increase hepatic PCSK9 levels [175]. This ‘statin paradox’ indicates that combinatorial use of PCSK9 inhibitors and statins has the potential to more effectively reduce LDL cholesterol in cardiovascular-risk patients [175,176].

In addition, PCSK9 has functions beyond the regulation of serum cholesterol levels that could be relevant for COVID-19 severity. PCSK9 blockage in rodent models of sepsis reduced inflammation and improved survival [177,178]. PCSK9 loss-of-function mutations were associated with better survival in sepsis, while high plasma PCSK9 levels or PCSK9 gain-of-function mutant carriers were described to contribute to disease severity and death [179,180]. In a further analysis, PCSK9 plasma levels were increased in patients with systemic inflammatory response syndrome and patients with sepsis in comparison to healthy controls but did not differ between severe and less severe cases. Accordingly, survival was not related to lower plasma PCSK9 levels [141]. On the other hand, PCSK9 loss-of-function variants did not protect from sepsis upon bacterial infection [181], and PCSK9 blockage did not lower the risk of severe infections and sepsis [182].

**Figure 4:**
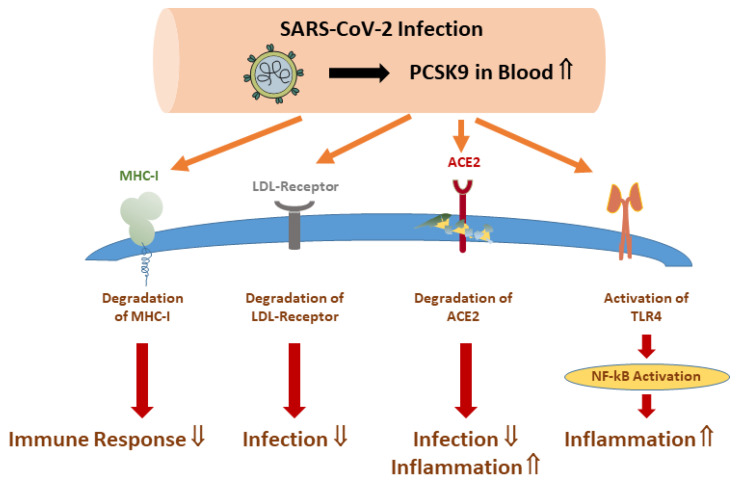
Role of PCSK9 in COVID-19 disease. Plasma/serum PCSK9 levels are elevated in SARS-CoV-2 infected patients. PCSK9 promotes degradation of ACE2 and the LDL receptor, which both can function as SARS-CoV-2 receptors. PCSK9 increases TLR4/NF-kB activity and promotes MHC-I degradation, which impairs the host immune response to viral infection. Abbreviations: ACE2, angiotensin-converting enzyme 2; MHC-I, major histocompatibility complex class I; NF-kB, nuclear factor kappa-light-chain-enhancer of activated B cells; TLR4, toll-like receptor 4; ⇓, reduction; ⇑, increase.

Cell-based studies revealed some of the regulatory circuits influenced by PCSK9. For instance, PCSK9 can activate TLR4 and NF-kB, and this pathway induced the expression of tissue factor and procoagulant activity in monocytes [183] (Figure 4). PCSK9 also promoted ACE2 degradation, which was more efficiently induced by the PCSK9 gain-of-function mutation D374Y [49], suggesting a role for PCSK9 in SARS-CoV-2 infections (Figure 4). Furthermore, PCSK9 impaired the recycling of major histocompatibility complex class I (MHC-I) and favoured its lysosomal degradation [184] (Figure 4). As MHC-I expression is suppressed upon SARS-CoV-2 infection, PCSK9-mediated MHC-I downregulation may enable the virus to prevent CD8 + T-cell mediated clearance [185]. Thus, PCSK9 blocking antibodies may improve the host defence against SARS-CoV-2. Indeed, better outcomes of severely ill COVID-19 patients treated once with the PCSK9 inhibitor evolocumab were reported [186].

Statin-mediated PCSK9 upregulation [175,176] paved the way for studies that identified SREBP2 as inducing PCSK9 expression. Most relevant in the context of COVID-19 disease progression, SREBP transcription factors are activated upon SARS-CoV-2 infection [49,142,187]. In this manner, peripheral blood mononuclear cells of COVID-19 patients displayed increased PCSK9 mRNA levels, and a modest association with disease severity was also noticed [188].

In patients with sepsis, SARS-CoV-2 infection caused an increase in plasma PCSK9 levels in contrast to sepsis patients lacking a viral infection [141]. Notably, there was a strong induction of systemic PCSK9 levels in patients with moderate COVID-19 disease that did not increase much further in severe COVID-19 cases. Thus, SARS-CoV-2 -induced PCSK9 upregulation appears rather unrelated to disease severity and is likely mediated by SREBP2 [188,189]. As mentioned above, extracellular PCSK9 enhanced ACE2 degradation [49] and vice versa: low PCSK9 levels may be related to higher ACE2 levels, which have anti-inflammatory activities but have also been associated with increased virus infection. However, data on a potential role for PCSK9 antibodies interfering with PCSK9-induced ACE2 degradation are still lacking [3,49].

A Mendelian randomisation study could not provide evidence for an association between PCSK9 expression and COVID-19 disease severity or outcome [190]. Accordingly, plasma levels of PCSK9 were not associated with in-hospital mortality in a cohort of 97 patients [19] and in 55 patients with severe COVID-19 [189]. The role of PCSK9 blockage as a therapeutic approach for severe COVID-19 thus needs further study.

## 9. Novel Drugs to Treat Dyslipidaemia and COVID-19 Disease

Besides statins, ezetimibe and antibodies targeting PCSK9 listed in the previous sections, several novel drugs to treat dyslipidaemia have been developed [191]. This includes the small interfering RNA molecule inclisiran, which lowers hepatic PCSK9 levels and exhibits significant promise. Pelacarsen is an antisense oligonucleotide that targets and reduces lipoprotein (a) levels. In addition, volanesorsen represents the first medication to target chylomicrons and lower triglyceride levels. In this context, olezarsen, an antisense oligonucleotide targeting apoCIII, has shown promise to lower chylomicron levels [191]. A monoclonal antibody which inhibits angiopoietin-like 3 protein (evinacumab) is used for the treatment of familial hypercholesterolaemia [191]. As these medications only became available recently, the effects of these novel cholesterol- and triglyceride-lowering drugs for SARS-CoV-2 infection, disease severity and outcome have yet to be examined.

Currently, the only available information on the impact of a novel cholesterol-lowering treatment on COVID-19 disease outcome exists for the oral adenosine triphosphate citrate lyase inhibitor bempedoic acid. This drug decreases plasma LDL-cholesterol levels by lowering hepatic cholesterol synthesis and simultaneously increasing LDL clearance due to upregulated LDL-receptor expression. Approximately 14,000 patients randomly assigned to receive bempedoic acid or placebo in a double-blind trial with a median follow-up of 3.4 years showed bempedoic acid to efficiently reduce LDL cholesterol of statin-intolerant patients [192]. However, prevalence of COVID-19 and COVID-19 pneumonia did not differ between these two groups [193].

## 10. SARS-CoV-2 Infection Modulates Cholesterol-Sensitive Pulmonary Functions

The lungs are the primary target of SARS-CoV-2, and mild to severe lung damage may occur upon infection [194,195]. However, although lipids have been increasingly linked to lung diseases, there is still limited knowledge on the role of cholesterol metabolism in proper lung functioning. Yet, studies outlined below highlight that cholesterol homeostasis is critical for pulmonary function and immune response in COVID-19.

Statins were recently described to improve lung function of patients with chronic obstructive pulmonary disease, further suggesting a role for cholesterol-regulated pathways herein [196]. Indeed, earlier studies identified reduced amounts of HDL cholesterol and ApoA-1 to be associated with interstitial lung disease [197]. This phenotype was proposed to reflect the role of HDL in reverse cholesterol transport, which refers to ApoA-1 and HDL serving as acceptors for excess cellular cholesterol, followed by their transport to the liver for bile acid synthesis and secretion [84]. The transfer of cellular cholesterol onto extracellular ApoA-1 is mediated by the ATP-binding cassette transporter A1 (ABCA1) [84], and ABCA1 mutant mice showed pulmonary cholesterol overload along with the appearance and accumulation of cholesterol-loaded macrophages (foam cells) in the lung [198]. In other studies, these foam cells were shown to arise early in pulmonary diseases to contribute to lung fibrosis [199]. Supporting the requirement to remove excess pulmonary cholesterol, ApoA-1-deficient mice revealed an exacerbated pulmonary neutrophil response after LPS inhalation, while administration of recombinant ApoA-1 or HDL attenuated acute lung injury [164,200,201].

Macrophages and neutrophils were enriched in bronchoalveolar lavage in severe compared to moderate COVID-19 [202], and single-cell RNA sequencing revealed an upregulation of the G-protein coupled receptor GPR183, initiating migration of macrophages towards a gradient of the oxysterol 7α,25-dihydroxycholesterol in COVID-19 patients [203,204]. This was accompanied by a higher expression of cholesterol 25-hydroxylase and cytochrome P450 family 7 subfamily member B1, which both contribute to the production of 25-hydroxycholesterol and 7α,25-dihydroxycholesterol [204]. Dysfunctional GPR183 and the GPR183 antagonist NIBR189 efficiently reduced macrophage infiltration into the lungs and inflammatory cytokine expression in SARS-CoV-2-infected mice. Moreover, GPR183-deficient mice exhibited less severe disease upon infection with SARS-CoV-2 and oral NIBR189 administration enabled a faster recovery after SARS-CoV-2 infection compared to vehicle-treated controls [204].

While the study implicated harmful effects of elevated oxysterol levels in COVID-19, others reported rather protective activities of 25-hydroxycholesterol in lung inflammation [204,205]. Mice lacking cholesterol 25-hydroxylase had delayed resolution of neutrophilia after LPS inhalation, which could be improved by systemic administration of 25-hydroxycholesterol. In support of the latter findings, mice lacking liver X receptor (LXR), a transcription factor binding and facilitating oxysterol-induced changes in gene expression [206], also exhibited worse lung disease. LXR agonists are well-known to promote reverse cholesterol transport [207], which, as outlined above, could resemble a protective mechanism in lung inflammation [164,200,201], and indeed, LXR agonists accelerated the resolution of inflammation, indicating a therapeutic potential for 25-hydroxycholesterol and other LXR agonists in diseases associated with pulmonary inflammation [205].

In support of the latter, serum concentrations of 25-hydroxycholesterol were slightly lower in severe COVID-19 compared to healthy controls. The serum levels of 27-hydroxycholesterol, a further metabolite produced from cholesterol by enzymatic processes and shown to exert antiviral activity, was strongly reduced in SARS-CoV-2 infection and declined with disease severity [131]. As proposed by others [208], the decline in oxysterols with an enzymatic origin may in part be related to mitochondrial dysfunction and impaired activity of mitochondrial 27-cholesterol hydroxylase. In contrast, 7-ketocholesterol and 7 β-hydroxycholesterol, which are formed by the auto-oxidation of cholesterol, were increased in the serum of patients with moderate and severe COVID-19 [131,208]. Hence, as oxysterols with a nonenzymatic origin are considered markers of oxidative stress, their increase in SARS-CoV-2 infection appears plausible [208]. Interestingly, antiviral SARS-CoV-2 activities were shown for both enzymatic (27-hydroxycholesterol) and nonenzymatic oxysterols (7-ketocholesterol) in cultured cells [209].

Other antiviral effects of 25-hydroxycholesterol have been reviewed in detail [3,26] and might also be relevant to ameliorate lung disease in COVID-19. This includes 25-hydroxycholesterol reducing cholesterol availability at the cell surface and interfering with the fusion of SARS-CoV-2 with the plasma membrane or triggering cholesterol accumulation in endolysomes and compromising S protein-dependent fusion with endolysosomal membranes. Furthermore, 25-hydroxycholesterol might compromise pulmonary viral entry and propagation through inhibition of SREBP2, thereby (i) reducing the production of cholesterol precursors that ultimately restrict glycosylation and prenylation of proteins, (ii) compromising cholesterol availability to ensure membrane raft integrity and (iii) activating LXR, thereby lowering cellular cholesterol levels through upregulated cholesterol efflux [26].

In addition to dysregulated cholesterol export pathways possibly contributing to pulmonary dysfunction in COVID-19, lipoproteins delivering cholesterol and triglycerides are also essential for pulmonary function. In this way, surfactant lipids are important for lung function and contain 5–10% cholesterol, which is elevated in patients with chronic lung diseases [210]. In addition, ApoE-deficient mice, which are characterised by increased LDL and VLDL levels, displayed an abnormal surfactant lipid composition, suggesting these lipoproteins to be crucial for a healthy lung [201].

As mentioned above, statins improve lung function in chronic obstructive pulmonary disease [196], implicating the beneficial actions of statins to directly reduce inflammation and fibrosis in the lungs [3]. However, given the complex and diverse mechanisms that contribute to lung injury and cell death in patients with COVID-19, it remains difficult to pinpoint the particular advantageous therapeutic statin actions. Simvastatin was found to have no effect on acute respiratory distress syndrome (ARDS). However, patients with a hyperinflammatory ARDS phenotype had improved survival with simvastatin therapy compared with placebo [211]. Statins were also associated with a reduced likelihood of persistent cough and dyspnoea in diabetic patients with COVID-19. In addition, statins may also help to reduce the development of lung fibrosis associated with COVID-19 in patients with long-term diabetes [168]. Despite these observations, research on statin-mediated effects on lung cells remains limited. One study showed that simvastatin reduced inflammation in lung tissue and lung epithelial cells [146]. However, different statins did not impact the innate immune response of lung epithelial cells during SARS-CoV-2 infection. Fluvastatin significantly reduced viral protein translation and replication of SARS-CoV-2-infected differentiated human primary bronchial epithelial cells [212]. SARS-CoV-2-induced disruption of the lung endothelial barrier was ameliorated by fluvastatin [213]. Further studies will need to dissect the protective effects of different statins in the lung and to identify patients who may profit from statin therapy. It should be noted that statin-induced lung injury is a very rare adverse effect of these drugs [214].

Altogether, cholesterol homeostasis is essential for pulmonary function [201] and is affected by viral infections with pathophysiological consequences, providing therapeutic opportunities to modulate COVID-19 severity.

## 11. Conclusions

In this review, we summarised current knowledge on the potential association of serum cholesterol levels with COVID-19 severity. In many studies, a reduction in serum cholesterol levels was observed in patients with severe COVID-19. However, such changes have not been described in several other cohorts, and the discordance of these findings have not been finally clarified. There is evidence that the lowering of cholesterol levels is not associated with common clinical biomarkers of liver injury. Nevertheless, diagnosing hepatic injury in severe cases of COVID-19 remains a challenge. Increased activities of aminotransferases may indicate the presence of inflammation rather than liver damage. While not universally corroborated, the overall evidence suggests that patients undergoing statin therapy or initiating statin therapy during SARS-CoV-2 infection may exhibit a reduced severity of disease. In addition to their cholesterol-lowering effects, statins appear to exert additional beneficial mechanisms that further contribute to the overall protective effects of these drugs. Alternatively, the encouraging results of a recent study on the protective effects of PCSK9 inhibition in patients with severe SARS-CoV-2 infection may initiate the conduct of larger clinical trials.

## Figures and Tables

**Table 1:** Current knowledge on the association of cholesterol with COVID-19 severity. The table summarises the still inconsistent results for (i) the association of dyslipidaemia before SARS-CoV-2 infection and COVID-19 outcome, (ii) the association of blood cholesterol with disease severity and outcome and (iii) the effect of statin therapy on SARS-CoV-2 infection and outcomes. The references related to these observations are provided.

Studies Showing Cholesterol-Related Associations with COVID-19 Severity	Studies Lacking Cholesterol-Related Associations with COVID-19 Severity
(i) Dyslipidaemia before infection increased the risk for SARS-CoV-2 infection [9,10].	(i) Dyslipidaemia before infection was not associated with the risk for SARS-CoV-2 infection [11].
(i) Dyslipidaemia before infection increased the risk for a severe disease course and mortality [9,10,12,13].	(i) Dyslipidaemia before infection was not associated with the risk for a severe disease course and mortality [14].
(ii) Low blood cholesterol in patients with COVID-19 was related to a severe disease course and mortality [15,16,17].	(ii) Blood cholesterol in patients with COVID-19 was not related to a severe disease course and mortality [18,19,20].
(iii) Preadmission statin treatment was associated with better outcomes among COVID-19 patients [21,22,23].	(iii) Statin use before COVID-19 hospitalisation did not protect from fatal outcomes [24].
